# Two-trocar appendectomy in children – description of technique and comparison with conventional laparoscopic appendectomy

**DOI:** 10.1186/s12893-016-0170-1

**Published:** 2016-08-04

**Authors:** Martin Salö, Emil Järbur, Mette Hambraeus, Bodil Ohlsson, Pernilla Stenström, Einar Arnbjörnsson

**Affiliations:** 1 Department of Clinical Sciences, Pediatrics, and Department of Pediatric Surgery, Lund University, Skåne University Hospital, Lasarettsgatan 48, Lund, 221 85 Sweden; 2 Department of Clinical Sciences, and Division of Internal Medicine, Lund University, Skåne University Hospital, Malmö, 205 02 Sweden

**Keywords:** Acute abdomen, Appendicitis, Appendectomy, Children, Laparoscopy

## Abstract

**Background:**

The aim of the study was to describe the technique of two-trocar laparoscopic appendectomy and compare the outcome between two- and three-trocar techniques in children.

**Methods:**

All children who underwent laparoscopic surgery for suspected appendicitis from 2006 to 2014 in a center for pediatric surgery were included in the study. Converted surgeries and patients with appendiceal abscess or concomitant intestinal obstruction were excluded. A total of 259 children underwent appendectomy with either two (35 %) or three (65 %) laparoscopic trocars according to the surgeons’ preference and intraoperative judgment. Patient demographics, clinical symptoms, surgery characteristics, and complications were reviewed.

**Results:**

The mean age of the children was 10.4 years (range, 1–14 years). The mean follow-up time was 41.2 months (SD ± 29.2). No significant differences in age, gender, weight, or signs and symptoms were found between the two- and three-trocar groups. The mean surgery time was significantly shorter in the two-trocar group (47 min) than in the three-trocar group (66 min; *p* < 0.001). The rates of surgical complications were 2 % vs. 4 %, (*p* = 0.501), and the rates of postoperative complications were 0 % vs. 5 % (*p* = 0.054), in the two- and three-trocar groups. The overall incidence of postoperative wound infection was low (<1 %) and did not differ between groups.

**Conclusions:**

Two-trocar laparoscopic appendectomy seems to be a safe and feasible technique with a low rate of postoperative wound infections. The present findings demonstrate that when the two-trocar technique could be applied, it is a good complement to the conventional three-trocar technique.

## Background

Appendicitis is the most common abdominal disease that requires surgery in children [1–5] . Most studies show that laparoscopic appendectomy (LA) has advantages over open appendectomy (OA) in children [6–8]. Publications also claim that intravenous antibiotics may serve as monotherapy for acute appendicitis [9, 10], but long-term results of these findings are lacking. Nonetheless, appendicitis is for sure, cured through appendectomy.

Since Kurt Semm described a technique for endoscopic appendectomy in 1983 [4], several surgical techniques for LA have been described. Currently, the technology for performing appendectomy utilizes one, two, three, or more trocars [11–14]. The two-trocar LA is a result of trying to overcome two major disadvantages of three-trocar LA when it originally was compared to OA; longer operative time and greater cost [15, 16]. Two-trocar laparoscopic-assisted appendectomy has been described previously in children but without comparison with other techniques [13, 16]. In adults, two-trocar LA has been compared with OA and conventional three-trocar LA [17–20]. In our pediatric surgery clinic, the laparoscopic technique utilizes two trocars or conventional LA with three trocars. Some surgeons are concerned about the two-trocar LA and afraid of increased rates of wound infections compared to conventional LA [16, 17, 20], and one study added cleansing of the wound with peroxide for 1 week postoperatively [15]. Our hypothesis was that the two-trocar LA technique is as safe as three-trocar LA and does not increase the rate of wound infections. The aim of the present study was to describe the two-trocar LA technique in pediatric appendicitis and compare outcomes between two- and three-trocar techniques with regard to surgery time and complications, including the rate of postoperative wound infection.

## Methods

The regional research ethics committee approved the study (registration no. 2010/49). Parents were informed about the intent to perform a LA and about the risk of conversion to open appendectomy. No specified information about the two- or three-trocar LA was given. Since this was a retrospective study of performed LAs, no written consent was taken beforehand.

### Children and clinical data

#### Data collection

All children (<15 years of age) who underwent LA from January 2006 to December 2014 in the Department of Pediatric Surgery were retrospectively included in the study. Exclusion criteria were converted LAs, patients with an appendiceal abscess, and patients with concomitant intestinal obstruction. Data were retrieved from an electronic database of medical records.

Acute appendicitis was diagnosed based on clinical prediction scores. The diagnosis was occasionally assisted by ultrasound. The diagnosis of appendicitis was confirmed by surgical findings combined with the histopathological analysis. Age, gender, weight, preoperative work-up, and appendiceal grade of inflammation was recorded. The time interval from admission to the start of surgery was defined as the time interval from the decision that the child should be transferred from the emergency room to the start of the operation. Information about the surgical method used (i.e., two- or three-trocar technique) and the duration of surgery were collected from the surgical reports. Postoperative pain medication was recorded from the moment the child left the postoperative unit and arrived in the pediatric surgical ward. Postoperative pain management, operative and postoperative complications (including wound infection), and duration of long-term follow-up were recorded.

Six surgeons attended and were responsible for the LAs. All of the responsible surgeons were specialists in general surgery or pediatric surgery. The decision to perform LA using the two-trocar technique was based on the surgeons’ intraoperative judgment for the individual child. In children where there was no need for diathermy or scissors to perform the appendectomy, only two trocars were used. All of the surgical interventions were preceded by antibiotic administration according to a previously published method [9]. Postoperative antibiotics were given to patients with gangrenous or perforated appendicitis.

#### Statistical analysis

SPSS (Statistical Package for the Social Sciences) was used for the statistical calculations. A power calculation of the sample size was performed with the aid of a statistician [10]. A minimum of 200 patients were needed to show a difference with 80 % power at a 5 % significance level. Children with appendiceal abscess were excluded to collect groups that are more comparable. To obtain comparable groups for calculation of pain management, patients with a negative appendectomy and patients with complications were excluded. Fisher’s exact test was used for dichotomous variables, and Student’s *t*-test and the Mann–Whitney U-test were used for ranked results with and without a standard distribution, respectively. Values of *p* < 0.05 were considered statistically significant.

### Surgical techniques

The laparoscopy starts with the insertion of a 3- or 5-mm umbilical- or subumbilical trocar using an open access technique. It is intended for the insertion of 30-grade, 3- or 5-mm laparoscopic optics, which are used for diagnostic purposes. After having positioned the first trocar and collected some diagnostic information, the intraoperative findings enable the surgeon to choose in going on with two or three trocars.

### Two-trocar technique

**Fig. 1:**
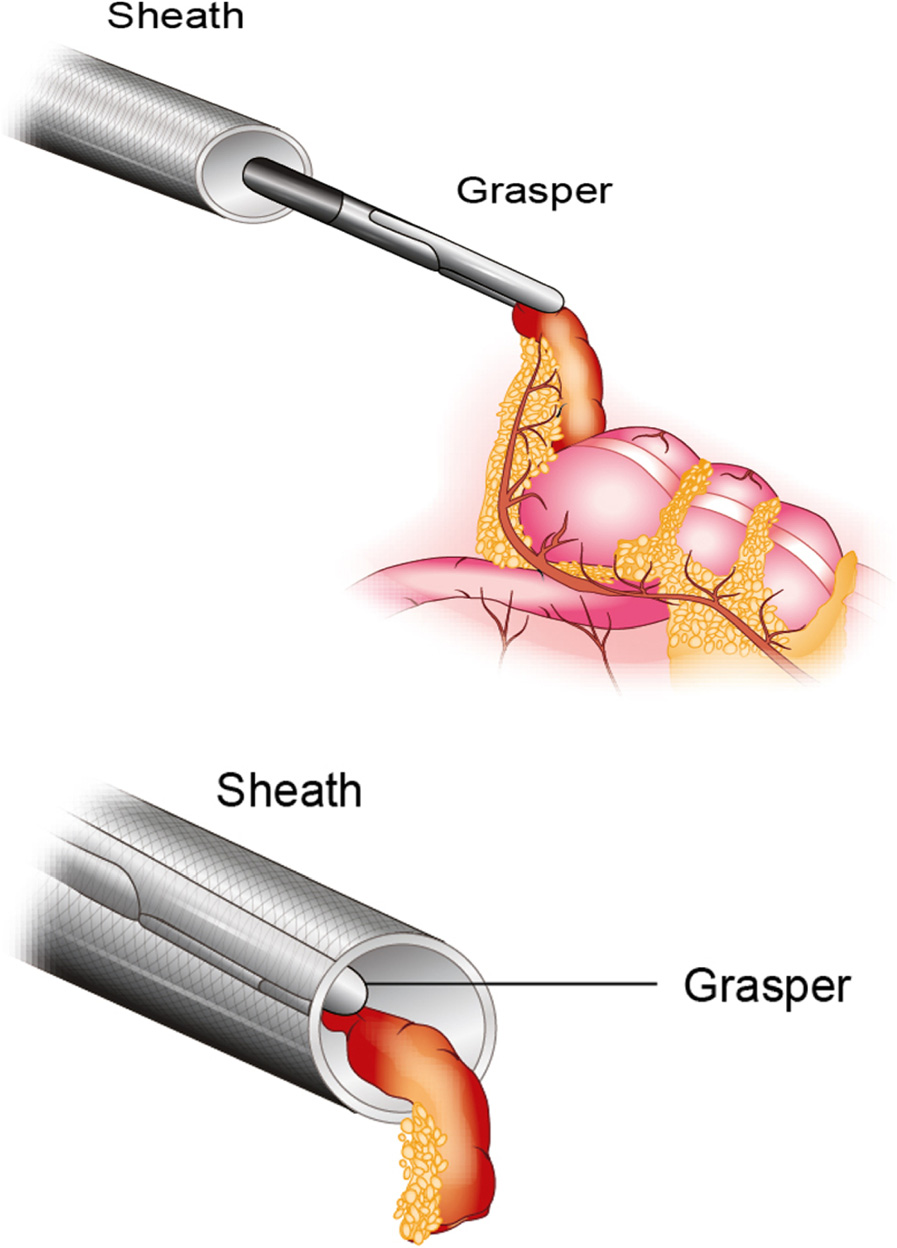
Picture describing two-trocar laparoscopic appendectomy. The appendix is visualized, grasped, and drawn into the Versa Step™ 12 mm trocar

If two-trocar LA is chosen, a Versa Step™ 10 or 12 mm trocar (provided by Covidien Autosutur™, Minneapolis, USA) is introduced into the abdominal cavity above the base of the cecum, which is located under direct vision through the laparoscopic optics and not necessarily at the point of maximum tenderness (McBurney’s point). The appendix is visualized. If there is suspicion of appendicitis, then it is grasped by a laparoscopic instrument (e.g., Maryland or Babcock) and drawn into the Versa Step™ 12 mm trocar (Fig. 1). The inner part of the trocar is drawn up from the sheath, thereby enfolding the appendix and holding it in place. Using this technique, the inflamed appendix never comes in contact with the tissue of the trocar hole in the abdominal wall. When the appendix is in the extracorporeal position, the grasper is substituted with a conventional Babcock. The sheath (acting as an endoscopic bag) is then gently withdrawn which exteriorizes the appendix removed. The pneumoperitoneum is slowly deflated when removing the trocar, which makes the exteriorization of the appendix easier. Electric cautery is used to divide and seal the vessels in the mesoappendix. The appendix is ligated at the base with an absorbable suture, leaving no metal staples in the growing body of the child. After removing the appendix, pneumoperitoneum is established again and the abdominal cavity is inspected, ensuring hemostasis. Finally, the abdominal gas is emptied, and the trocar wounds are sutured.

### Three-trocar technique

If three-trocar LA is chosen, two 5-mm trocars is placed; one at 1–2 in. above the symphysis and one to the left (usually in the left iliac fossa). The dissection of the mesoappendix is performed using an electrocautery hook, and the appendix is divided at the base with staples.

## Results

### Patient characteristics

During the studied period, 324 children underwent laparoscopic appendectomy. Of these, 56 children were excluded from the study because of conversion from three-trocar LA to open surgery. The reasons for these conversions were: perforated appendicitis with pus spread in the abdominal cavity (*n* = 42); anatomical difficulties making the laparoscopic technique unsafe (*n* = 10); and technical difficulties with the laparoscopic equipment (*n* = 4). No two-trocar LAs were converted to the conventional three-trocar technique or to open surgery. Further, six patients who had an appendiceal abscess diagnosed peroperatively and three patients with concomitant intestinal obstruction were also excluded, leaving totally 259 children included in the study. Of the children with an appendiceal abscess, two were operated on with three-trocar LA, one with two-trocar LA, and the other three were directly converted to open appendectomy. The clinical diagnosis was assisted by ultrasound in 37 % of the patients. Of the 259 children, 168 (65 %) underwent surgery with the conventional three-trocar technique, and 91 (35 %) underwent surgery with the two-trocar technique. There was no difference in demographics between the two groups (Table 1). The mean long-term follow-up was 38 months (range, 3–104 months) for children who underwent the three-trocar procedure and 47 months (range, 2–106 months) for children who underwent the two-trocar procedure (Table 1). An equal number of patients underwent surgery with the two-trocar technique each year during the study period.

**Table 1:** Descriptive data on children with suspected appendicitis who underwent either two- or three-trocar laparoscopic appendectomy

	Two-trocar LA (*N* = 91)	Three-trocar LA (*N* = 168)	*p-*value
Males/Females	50/41 (55/45)	94/75 (56/44)	1.000^c^
Age (years)	10.3 ± 3.3	10.5 ± 2.8	0.652^d^
Age group			
4 years	5 (5)	4 (2)	0.293^c^
5–9 years	24 (27)	47 (28)	0.882^c^
10–14 years	62 (68)	117 (70)	0.881^c^
Weight by age group (kg)			
4 years	15 (15–17)	16 (14–18)	X
5–9 years	27 (17–38)	27 (14–45)	0.966^d^
10–14 years	48 (24–72)	50 (26–87)	0.062^d^
Time from admission to appendectomy (h)	4 (1–41)	4 (0.5–34)	0.412^c^
Duration of symptoms (h)	24 (2–240)	24 (3–168)	0.314^e^
CRP value (mg/L)	12 (0–263)^a^	21 (0–365)^b^	0.071^e^
Leucocytosis	53 (58)	93 (55)	0.691^c^
Fever > 38 °C	32 (35)	71 (42)	0.294^c^
Grade of inflammation			
Phlegmonous	56 (92)	114 (68)	0.341^c^
Gangrenous	9 (10)	21 (13)	0.682^c^
Perforated	5 (5)	14 (8)	0.462^c^
Negative appendectomy	21 (23)	19 (11)	0.023^c^
Long-term follow-up (months)	47 ± 30	38 ± 28	0.023^d^

Values are given as: *n* (%) = absolute number and percentage of patients; mean ± standard deviation (SD); or median (min–max); X = too few patients

*CRP* C-reactive protein *LA* laparoscopic appendectomy

^a^ Three patients with missing data

^b^ Seven patients with missing data

^c^ Fisher’s exact test

^d^ Students *t*-test, two-tailed

^e^ Mann–Whitney U –test, two-tailed

With regard to the duration of symptoms, time to appendectomy, presence of fever or leukocytosis, and grade of inflammation, no significant differences were found between the two groups. However, a significantly higher rate of negative appendectomies was found in patients who underwent two-trocar LA (Table 1).

### Evaluation of the surgical technique

The surgery time with the two-trocar technique was significantly shorter than with the three-trocar technique, both after inclusion and exclusion of negative appendectomies (Table 2).

The few surgical complications were iatrogenic perforations (*n* = 7), bleeding (*n* = 1), and diathermic injury (*n* = 1), which did not differ between groups. Further, no difference in the rate of postoperative complications, excluding wound infections, was observed between the two groups. The complications were postoperative abscess (*n* = 6) and postoperative intestinal obstruction *n* = 2). The rate of postoperative wound infection was low (<1 %) and did not differ between groups (Table 2).

**Table 2:** Differences in surgery time and complications between two- and three-trocar laparoscopic appendectomies

	Two-trocar LA (*N* = 91)	Three-trocar LA (*N* = 168)	*p-*value
Surgery time all included (min)	47 ± 16	66 ± 22	<0.001^e^
Surgery time with negative appendectomies and patients with surgical complications excluded (min)	46 ± 16	65 ± 20	<0.001^e^
Excluded patients	23 (25)	26 (15)	
Surgery time > 60 min	12 (13)	86 (51)	<0.001^d^
Surgery time in patients with surgical complications (min)	46 ± 1	81 ± 20	0.003^e^
Included patients	7 (8)	2 (1)	
Number of surgical complications	2^a^ (2)	7^b^ (4)	0.501^d^
Number of postoperative complications	0 (0)	8^c^ (5)	0.054^d^
Wound infection	1 (1)	1 (1)	1.000^d^

Values are given as: *n* (%) = absolute number and percentage of patients; mean ± standard deviation (SD); or median (min–max)

*LA* laparoscopic appendectomy

^a^ Two iatrogenic perforations

^b^ Five iatrogenic perforations, one postoperative bleeding, and one diathermic injury

^c^ Six patients with postoperative abscess and two patients with postoperative intestinal obstructions

^d^ Fisher’s exact test

^e^ Student’s t-test, two-tailed

No significant differences were found between the two groups in the postoperative use of morphine, paracetamol, or nonsteroidal anti-inflammatory drugs (NSAIDs) (Table 3).

**Table 3:** Postoperative pain management

	Two-trocar LA (*N* = 68)	Three-trocar LA (*N* = 135)	*p-*value
Morphine administration	15 (22)	37 (27)	0.502^b^
Total amount of morphine^a^ (mg/kg)	0.11 ± 0.09	0.11 ± 0.09	0.744^c^
NSAID administration	38 (57)	73 (54)	0.772^b^
Single doses	25 (37)	45 (33)	0.644^b^
Regular treatment	13 (20)	28 (21)	1.000^b^
Paracetamol intravenously (doses)	2 (0–10)	2 (0–18)	0.914^d^

Negative appendectomies and patients with surgical and postoperative complications excluded. Values are given as: *n* (%) = absolute number and percentage of patients; mean ± standard deviation (SD); or median (min–max)

*LA* laparoscopic appendectomy, *NSAID* nonsteroidal anti-inflammatory drugs

^a^ In patients receiving morphine

^b^ Fisher’s exact test

^c^ Student’s *t*-test, two-tailed

^d^ Mann–Whitney U-test, two-tailed

Advantages and disadvantages of two- and three-trocar LAs are summarized in Table 4.

**Table 4:** Advantages and disadvantages of two- *vs*. three-trocar laparoscopic appendectomy

	Two-trocar LA	Three-trocar LA
Advantages	• Less trauma • Only two scars on the abdomen • Shorter surgery time • Cheaper • Shorter learning curve	• More instruments in the abdomen • Diathermy • Can be used with adhesions or retrocecal appendix • More often applicable
Disadvantages	• Only one instrument • Cannot use diathermy • Limited mobility in the abdominal cavity and less able to explore the intestinal package • Cannot get traction to resolve adhesions • Not always applicable	• Longer surgery time • More scars • More trauma

*LA* laparoscopic appendectomy

## Discussion

This was the first study with comparison of two-trocar LA with another appendectomy method in children (Table 5). Based on the study results presented here, we accepted the research hypothesis that two-trocar LA was a safe and quick technique with a low rate of postoperative wound infection.

**Table 5:** Overview of studies of two-trocar LA in children and adults

Study	Age group (N)	Trocar placement	Results
Valioulis et al. [13] No comparison.	Children (38)	Umbilicus and pubic symphysis	Success: 76 %, mean operation time 19 min, wound infection 3 %.
Tekin and Kurtoglu [16] No comparison.	Adults (440)	Umbilicus and McBurney	Success: 67 %, mean operation time 46 min, wound infection 4 %.
Konstadoulakis [17] Comparison with conventional LA.	Adults (37)	Left iliac fossa and McBurney	Success 80 %, mean operation time 48 min, wound infection 11 %.
Malik et al. [18] Comparison with OA	Adults (14)	Umbilicus and McBurney	Success 11 %, mean operation time or wound infection not specified for two-trocar LA only.
Yagnik et al. [21] Comparison with OA and conventional LA.	Adults (61)	Umbilicus and Mcburney	Success 100 %, mean operation time 36 min, wound infection 1 %.
Baid et al. [20] Comparison with conventional LA	Adults (38)	Umbilicus and Mcburney	Success 84 %, mean operation time 24 min, wound infection 16 %

*LA* laparoscopic appendectomy

### Wound infection

One fear surgeons have with the two-trocar technique is that it may result in a higher rate of wound infections, since the inflamed appendix comes in contact with all the layers of the abdominal wall and the skin when it is being drawn out [16, 17, 20]. However, with our technique, the outer part of the trocar enfolds the inflamed appendix, which prevents contact with the abdominal wall. Accordingly, the rate of wound infection in the present study was very low (1 %). Previously studies have reported rates between 1 and 16 % [13, 16, 17, 20, 21]. Diverse types of trocars may explain these differences.

### Surgery time

The mean duration of two-trocar LA in the present study was similar to some reports [16, 17], but longer compared to others [13, 20, 21]. The shorter surgery time in the two-trocar group compared to the three-trocar group may be due to selection bias, hence the two groups were non-equivalent since the decision about which kind of procedure performed depended on the surgeon. We presume that each surgeon in this study used the surgical method that was the most beneficial for the children according to the own experience, and selected children for whom two-trocar LA could be applied.

### Feasibility and advantages with the two-trocar LA

In the present study, none of the patients who initially underwent two-trocar LA were converted to three-trocar LA or open appendectomy. To a certain extent, this may be explained by selection bias mentioned above. In previous studies, the reported rate of successfully completed two-trocar surgeries was 11 % [18], 67 % [16], 76 % [13], 80 % [17], 84 % [20] and 100 % [21]. From the present and previous studies, it is impossible to draw the conclusion that the shortened surgery time per se could be related to a lower complication rate. However, the two-trocar technique gives the surgeon more control over bleeding when dividing the mesoappendix. Also, less instruments are used and no staples are required, also reducing the cost. Since appendicitis is common, a small change in outcome can have major effect for the resources and costs of the health care system.

One limitation of the two-trocar technique is that it is difficult or impossible to use in a child with appendicitis and adhesions or retrocecal appendix [17]. Further, it may not always be advisable to grasp and pull an inflamed appendix, which can be fragile and tortuous. This decision has to be based on the intraoperative judgment of the surgeon. Hence, if there is no need for diathermy or scissors to perform the appendectomy, a third trocar seems unnecessary. The technique described can be applied for fat patients with ample adipose tissue, but hardly for extremely fat patients. Nevertheless, the technique has been described in adults before in which the abdominal wall is much thicker compared to children. From this study, it seems that there may be three factors that influence which technique the surgeon chooses: 1) The anatomical position of the appendix. If the appendix has a distinct retrocecal direction, the two-trocar technique may be more difficult; 2) adhesions that fixate the appendix that only can be dissected with the help of cautery; and 3) the preference of the surgeon, hence experience with the method. Regarding the last factor, we consider the two-trocar LA, compared to conventional LA, to be easier to learn for the young surgeon. In summary, this leads us to conclude that because of assumedly shorter learning curve compared to single-port LA [22], faster surgery time [17], shortened anesthesia, less trauma and reduced costs compared to conventional LA [23], and being a safe procedure; two-trocar LA has a role among different laparoscopic techniques of appendectomy.

No definitive conclusions can be drawn regarding which of the two laparoscopic techniques is best, and under what circumstances each technique should be used, until a prospective, randomized study in children is conducted. However, the data presented in this report certainly add information that we can use in our daily practice. If we perform the two-trocar technique, it may benefit the child and the health care itself with reduced costs [23].

### Two-trocar LA vs. single-port LA

Many studies have described the technique of single-incision LA (SILA) or single-port LA (SPLA). We agree that SILA/SPLA is interesting and we recently published an article about this technique [24]. But, SILA/SPLA has also been shown to result in longer operative time, higher analgesic consumption, and greater hospital charges in children when a meta-analysis of RCT’s was performed [12]. Another meta-analysis in children found shorter length of hospital stay, but higher conversion rate, higher surgical difficulty, and higher hospitalization costs compared with conventional LA [25]. Quie et al. [26], concluded that “..there is no indication to use this approach over standard laparoscopic appendectomy”. Further, special instruments and longer learning curve are two other disadvantages of SPLA/SILA. Many appendectomies are performed by junior doctors/residents which have a harder time dealing with instrument collision, reduced operative work space, inadequate retraction and compromised view in SPLA/SILA [11]. As stated in Table 4, one disadvantage of the two-trocar LA, is the reduced ability to explore the abdominal cavity, especially when examining the small intestine. However, this is also stated when talking about the SILA/SPLA [27]. Together with the other advantages of the two-trocar LA mentioned before, we therefore believe there is an obvious role for the two-trocar LA among appendectomy techniques.

### Study limitations

As mentioned above, the main weakness of the study is that the decision about which kind of procedure performed depended on the surgeon. Hence, an obvious selection bias may be evident and the two groups are non-equivalent. A second weakness of the study is that it was retrospective, and not randomized. When we now know that the two-trocar laparoscopic appendectomy does not result in more complications including wound infection, a prospective, randomized study can be started. At last, there was an unequal number of patients in the two groups compared. However, the only way this influences the statistical calculations is that the prerequisite to detect a, in beforehand given/hypothesized, difference is greater when the groups are equal in number (highest power). Hence, having different number of patients in the groups does not influence the statistical calculations and the conclusions drawn in this study.

## Conclusions

Two-trocar LA seems to be a safe and feasible technique in children with a low rate of postoperative wound infections, and the present findings demonstrate that it could be considered as a good and safe complement to the conventional three-trocar technique. Future research will determine which method is the best treatment. Until then, the minimalized method described herein may be a good option.

## Abbreviations

LA, laparoscopic appendectomy; NSAID, non-steroidal anti-inflammatory drug; SD, standard deviation
